# 

**Published:** 1921-06-04

**Authors:** 


### St. George's Hospital.

The report presented at the annual meeting of St. George's Hospital, over which Mr. F. J. Frankau, the Deputy Treasurer, recently presided, states that the ordinary expenditure exceeded the ordinary income by £35,753. That deficiency has had to be met by utilising the legacies received in cash during the year, by the sale of stock, which realised £20,103, and by the sale of a house at Bexhill for £1,897. The fact that the cost of maintaining the hospital has increased from £40,358 in 1913 to £85,626 in 1920 makes it necessary to raise an additional amount of £30,000 per annum. The result of the special appeal early in the year was disappointing. New or additional subscriptions received in response to that appeal amounted to £288, donations totalled £5,586, and about £750 additional was obtained by means of collecting-boxes.

### Registration of Charities.

As the result of an inquiry instituted by the National Council of Social Service, a report on the registration of charities has been issued, in which it is recommended that, subject to certain qualifications, compulsory registration, following generally the lines of the War Charities Act, 1916, should be extended to all charities.

### The National Relief Fund.

### DISBURSEMENT OF SEVEN MILLION POUNDS.

THE final report of the National Relief Fund, formerly known as the Prince of Wales' Fund, has been issued, and with the exception of a comparatively small balance which has been allocated, the whole of the enormous amount of £6,975,124 has now been expended. Naturally the chief relief given was in respect of Service or semi-Service objects, such as helping the families of soldiers and sailors, close on two millions, for instance, having been distributed through the Soldiers' and Sailors' Families Association. There were, however, many sections of the community which were not affected quite so immediately by the war, and the agencies through which they were reached are all detailed in the accounts. The larger of the sums so given are explained in the body of the report. Professional classes, who were hit as badly as any by war conditions, have been reached through different organisations, and we notice among other grants £10,000 to the Nation's Fund for Nurses. The Relief Fund was many times asked to assist voluntary hospitals, but so long as the war continued, and it was impossible to estimate the ultimate demands, the Committee did not feel justified in acceding to this request, although they recognised that these institutions had been gravely prejudiced. But in view of the gravity of the situation as regards hospitals which arose last year it was, after consulting with the Minister of Health, decided to appropriate £700,000 towards meeting the war deficits arising during the five years ending December 31, 1918.

It will be noticed that the hospitals of the British Isles received as near as possible 10 per cent. of the sum administered by the Fund, and this treatment, it must be agreed, was exceedingly generous. The sum allocated was divided as follows: England and Wales, 80 per cent.; Scotland, 11 per cent.; Ireland, 9 per cent. As regards the provincial hospitals in England and Wales, the grants were sufficient to cover 90 per cent. of the difference between the aggregate ordinary income, including legacies, and the aggregate ordinary expenditure for the five years to which the grant relates. London, which had received an emergency grant from King Edward's Hospital Fund, received approximately 75 per cent. of their deficits. A full list of the hospitals participating is given in the report, which is published by H.M. Stationery Office, at 3d. net.

### PASSING EVENTS AND TOPICS.

### Sanitary Inspectors' Qualifications in New Zealand.

EVIDENCE of the interest that is taken by the Dominions in English standards and methods of administration is given in the New Zealand Health Act, which we summarised in our issue of April 2. One of its provisions is that "after the commencement of the Act no person shall be appointed as sanitary inspector who is not the holder of a certificate from the Royal Sanitary Institute, or who is not the holder of such other qualification in lieu of such certificate as may be prescribed in that behalf by regulations under this Act." Examinations for the certificate referred to have been held in New Zealand since 1911, and eighty-four certificates have been issued.

### The Best-run Infant Welfare Centre.

AT the annual general meeting of the Jewish Infant Welfare Centre, St. George's-in-the-East, Dr. Mary Scharlieb eulogised the Centre as the best run and most successful one she knew, and emphasised the importance of ante-natal work. The great vitality shown by some people in recovering from illness was, she said, due to the conditions under which they were born. The low death-rate among Jewish children was attributable to the fact that they were breast-fed. It was most desirable, on medical grounds, that infants should not be separated from their mothers. The growing usefulness of the Centre is evidenced by the fact that the total of daily visits in 1920 was 1,140, as against 324 in 1918.

### Garden Fete at Clapham.

PRINCESS HELENA VICTORIA will attend a Garden Fête at the South London Hospital for Women, Clapham Common, on Friday, June 24, and will open a Sale of Work in aid of the funds. The Princess will be received by the Viscountess Cowdray. The Scots Guards Band will be in attendance, and concerts and other attractions will be provided. Tickets may be obtained from the Secretary at the hospital.

June 4, 1921] [Price Twopence

### The Hospital

**RATES OF SUBSCRIPTION** (post free, payable in advance).
Three months, 3/3; six months, 6/6; twelve months, 13/-. (Should the cost of printing and paper as well as increased postage, necessitate alteration of these rates, the period paid for will be reduced proportionately on unexpired subscriptions.) THE HOSPITAL "Index" to Volume LXIX., October 1920 to March 1921, is now ready, price 1/1 per copy, post free. Address The Manager, THE HOSPITAL, "The Hospital" Building, 28/29 Southampton Street, Strand, London, W.C. 2.

